# Comparison of oral health-related quality of life among endodontic patients with irreversible pulpitis and pulp necrosis using the oral health-related endodontic patient’s quality of life scale

**DOI:** 10.1007/s10266-024-01011-2

**Published:** 2024-10-01

**Authors:** Fadil Abdillah Arifin, Yuhei Matsuda, Nurhayaty Natsir, Takahiro Kanno

**Affiliations:** 1 Department of Oral and Maxillofacial Surgery, Shimane University Faculty of Medicine, 89-1 Enya-cho, Izumo, Shimane 693-8501 Japan; 2 Department of Conservative Dentistry, Faculty of Dentistry, Universitas Muslim Indonesia, Makassar, Indonesia; 3 Department of Conservative Dentistry, Faculty of Dentistry, Hasanuddin University, Makassar, Indonesia

**Keywords:** Endodontic treatment, Irreversible pulpitis, OHRQoL, Pulp necrosis, Quality of life

## Abstract

This prospective cohort study aimed to clarify differences in the longitudinal effects on oral health-related quality of life (OHRQoL) among patients undergoing endodontic treatment for irreversible pulpitis and for pulp necrosis, using a newly developed oral health-related endodontic patient’s quality of life (OHQE) scale. This study included 131 patients diagnosed with irreversible pulpitis and pulp necrosis. Comprehensive data regarding the patient’s background, medical history, and dental history were collected. The OHQE was administered three times to each patient: before and after endodontic treatment, as well as 2 weeks after endodontic treatment as a follow-up. Statistical analysis was performed using a linear mixed model for repeated measurements of changes in the OHQE score over time in cases of irreversible pulpitis and pulp necrosis. The patients consisted of 48 (36.6%) males and 83 (63.4%) females with a mean age of 36.2 (standard deviation, 12.6) years. Of these, 62 (47.3%) had irreversible pulpitis, and 69 (52.7%) had pulp necrosis. Intragroup comparisons showed an improvement in the OHQE scores over time in both groups (*p* < 0.001). Group comparisons revealed no significant differences at any time point. No interactions or changes over time were observed between the two groups. No difference in the improvement of quality of life after endodontic treatment was seen in the two disease groups, and both groups improved over time. However, patients’ expectations of receiving endodontic treatment remained unchanged after treatment. Therefore, dental providers should consider explaining the value of endodontic treatment to patients and address the measures that contribute to patient satisfaction.

## Introduction

Before resorting to tooth extraction, every possible attempt should be made to conserve the tooth, as tooth loss significantly affects an individual’s quality of life (QoL) [1]. Endodontic treatment can maintain tooth integrity and avoid the need for extraction [2]. The objective of endodontic treatment is to eradicate or prevent infections inside the root canal system. It has a success rate ranging from 70 to 95%. The rapid development of novel materials and processes has resulted in enhanced filling and preparation methods, thereby elevating the necessary benchmarks for endodontic treatment and enhancing its overall quality [3]. The short- and long-term consequences of endodontic treatment on patient satisfaction and QoL should be considered, given the increasing demand for endodontic treatment [4].

A previous prospective longitudinal study conducted 6 months after individuals received endodontic treatment revealed a statistically significant alteration in oral health-related QoL (OHRQoL). Additionally, mild enhancement of OHRQoL was observed by 1 month after treatment [5]. Another study reported significant statistical disparities in patients’ QoL before and after treatment, depending on the level of experience of the operator (specialist, postgraduate, or student) [6]. After endodontic treatment, more than 90% of patients at the Jordan University of Science and Technology clinic showed changes in taste perception, pain sensitivity, eating capacity, need for food temperature adjustment, self-awareness, nocturnal awakenings, meal interruptions, relaxation, and sleep initiation [7].

Endodontic treatment primarily focuses on intentional treatments, related diseases, and pulpal exposure [8, 9]. Irreversible pulpitis and pulp necrosis are two types of pulp disorders that require endodontic interventions. Failure to undergo endodontic therapy may cause a lesion to spread beyond the tooth apex, potentially leading to periapical disease [10]. Irreversible pulpitis is characterized by intense inflammation and severe symptoms, including prolonged and exacerbated discomfort, particularly while the person is in a supine position [11, 12]. This discomfort can manifest abruptly and is frequently accompanied by heightened sensitivity to temperature and sweetness, which can persist for an extended period. Thus, pain can be intense, may persist in response to temperature variations, can be spontaneous and diffuse, and may be felt in various orofacial regions [11, 12]. Symptomatic irreversible pulpitis is a significant pain-related problem that negatively impacts OHRQoL [13].

Pulp necrosis may result from the gradual advancement of pulpitis, wherein the fragile pulp tissue within the tooth perishes due to various factors, including trauma or severe bacterial infection [14]. Necrosis is commonly caused by untreated cavities, frequent invasive tooth procedures, and pathological ischemia of the dental pulp [15]. Typically, the initial indication of dental pulp necrosis is localized discomfort in the tooth or surrounding region due to inflammation. The intensity of pain varies from mild to moderate to severe, depending on the extent of the injury, accompanied by swelling and discomfort while chewing caused by compression of the nerve root at the base of the tooth [16]. A previous cross-sectional study showed that children with pulp necrosis experienced more pronounced adverse effects on their OHRQoL than did those with untreated carious lesions that did not involve pulp necrosis [17].

However, few studies have compared the effects of root canal treatment on these two pulp diseases in terms of QoL. The newly developed oral health-related endodontic patient’s quality of life (OHQE) scale comprises three subscales**—**physical, psychological, and expectations**—**encompassing 37 items. The OHQE has been verified as a reliable and valid scale and can be used to measure the OHRQoL in endodontic patients [18]. The OHQE scale employed in this study offers various benefits**,** such as the ability to evaluate QoL in endodontic patients experiencing physical and psychological symptoms associated with dental pain. Moreover, this novel scale can assess the expected results of endodontic treatment [18].

Therefore, this study aimed to evaluate and compare the OHRQoL in patients with irreversible pulpitis and pulp necrosis using a recently established OHQE scale.

## Patients and methods

### Patients

**Fig. 1:**
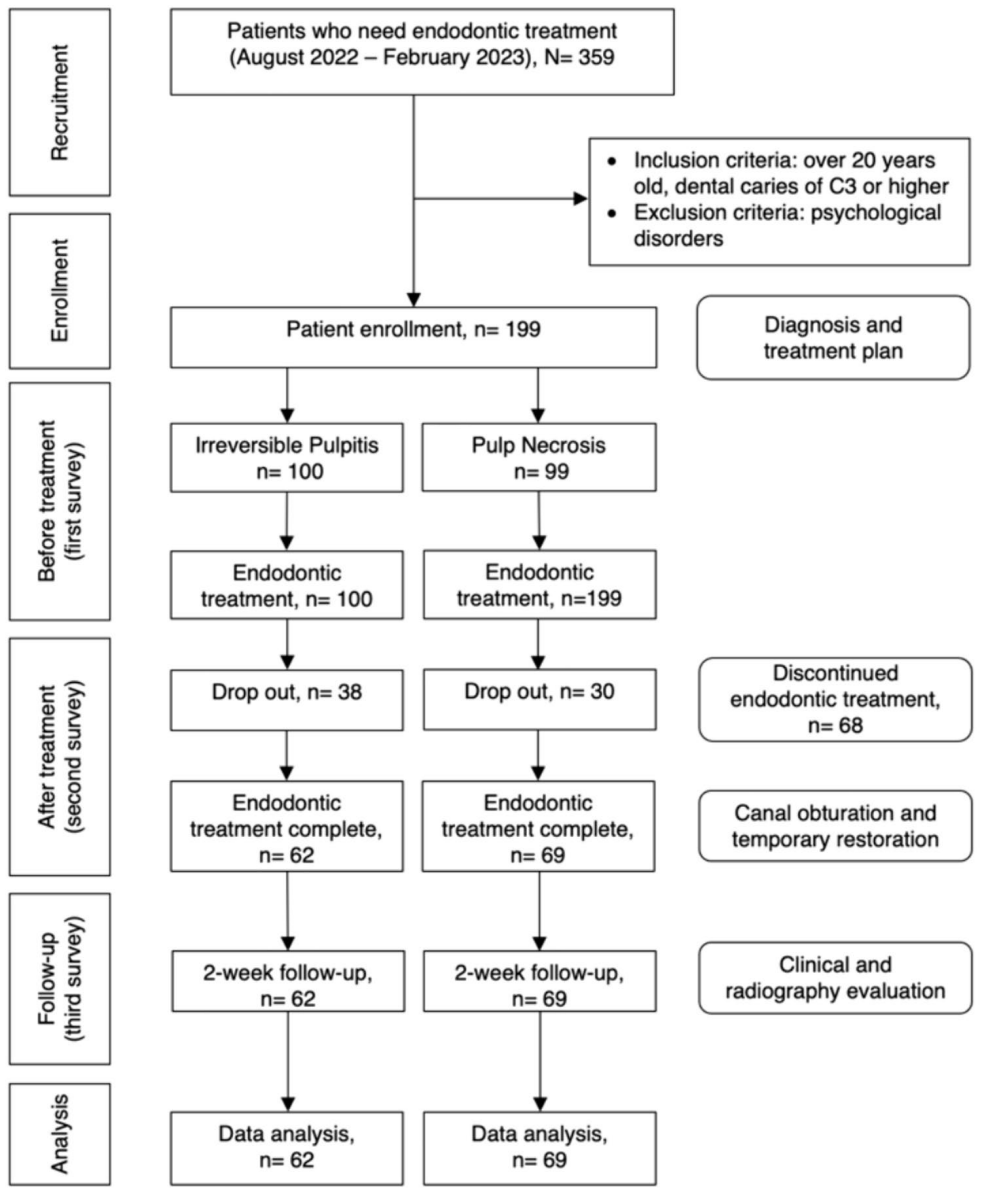
Flow-chart outlining the study

Between August 2022 and February 2023, 359 patients were directed to the Hasanuddin University Dental Hospital in Makassar, Indonesia, for endodontic treatment. In this prospective cohort study, patients who met the criteria of the study were recruited as respondents. The following inclusion criteria were set: (1) age > 20 years and (2) dental caries classified as C3 that penetrated the dental pulp or beyond, according to the International Caries Detection and Assessment System (ICDAS) caries classification [19]. Individuals with psychological problems were excluded from the study. Sixty-eight patients dropped out of the study due to discontinuing the treatment. Finally, 131 patients completed endodontic treatment, including the required follow-up. Of these patients, 62 had irreversible pulpitis, and 69 had pulp necrosis (Fig. 1). Each patient used the funds allocated by the Indonesian government’s National Health Insurance Program to finance their endodontic treatment expenses.

The OHQE scale was used to conduct three surveys. The first survey was conducted before the initiation of endodontic treatment. The second survey was conducted after the completion of the treatment and included root canal obturation and temporary restoration. The third survey in the current study was conducted 2 weeks later for a follow-up evaluation.

Informed consent was obtained from all individual participants included in the study. This study was performed in line with the principles of the Declaration of Helsinki. Approval was granted by the Ethics Committee of Universitas Muslim Indonesia and Ibnu Sina Hospital (Ethics approval number: UMI012206265). This study was conducted following the STROBE guidelines.

## Data collection

### Patient’s background data

We collected data from the patients regarding their age (years), sex, weight (kg), height (m), body mass index (kg/m^2^), employment status, university graduation, monthly income in Indonesian Rupiah (categorized according to Makassar City’s regional minimum wage: 3,300,000 IDR, equivalent to 210 USD), and number of housemates.

### Medical history

Medical history data, including the presence of systemic disease, medication use, and allergies, were obtained in this study.

### Dental history

We conducted a comprehensive dental examination on every individual to collect information regarding the overall tooth count, denture usage, grade of caries, grade of periodontal disease, frequency of daily brushing, and visual analogue scale (VAS) pain score, which ranged from 0 to 10. In this study, VAS was used as an instrument to determine the intensity of pain before and after endodontic treatment. The caries grade in the current study was classified according to the ICDAS: C3 (caries that penetrated the dental pulp) and C4 (the root of the tooth remained intact) [19]. Periodontal disease was categorized into four grades based on a new classification from the World Workshop on the Classification of Periodontal and Peri-implant Diseases and Conditions: S1, initial; S2, moderate; S3, severe and may result in tooth loss; and S4, severe and may result in the loss of all teeth [20].

### Diagnosis

The present study used the evaluation protocols recommended by the American Association of Endodontists to establish an endodontic diagnosis [21].

### Irreversible pulpitis

Irreversible pulpitis is diagnosed when both subjective and objective evidence confirms that the inflamed pulp cannot heal and that endodontic treatment is necessary. Common features include sharp pain in response to a thermal stimulus lasting for at least 30 s after removal of the stimulus, pain without any apparent cause, and pain referred to other teeth. These features are characteristic of symptomatic irreversible pulpitis. In contrast, asymptomatic irreversible pulpitis is characterized by the absence of noticeable clinical symptoms [22]. In this study, all patients diagnosed with irreversible pulpitis had pain symptoms, with an average VAS score of 4.5. Consequently, all of these patients were included as symptomatic irreversible pulpitis patients.

### Pulp necrosis

Pulp necrosis is a clinical diagnosis that refers to the death of the tooth pulp. Such pulp does not show any response to pulp testing and does not exhibit any symptoms. Calcification, recent trauma, or a lack of response may cause certain teeth to be unresponsive to pulp testing [22].

### Endodontic treatment

The endodontic treatment for all patients in this study was performed by endodontic residents of the Faculty of Dentistry at Hasanuddin University Dental Hospital. At every step of the procedure, the endodontic treatment follows standardized protocols and is supervised by a senior endodontist supervisor. This includes access opening, cleaning and shaping, and canal obturation steps. The utilization of tools and materials, including the type of anesthesia administered to the entire patient, adheres to the standardized endodontic treatment protocols. Thus, all patients in this study received endodontic treatment of relatively similar quality. In addition, all endodontic treatments were conducted in teeth with single and multiple canals, both maxillary and mandibular, during multiple visits.

### Questionnaire

**Fig. 2:**
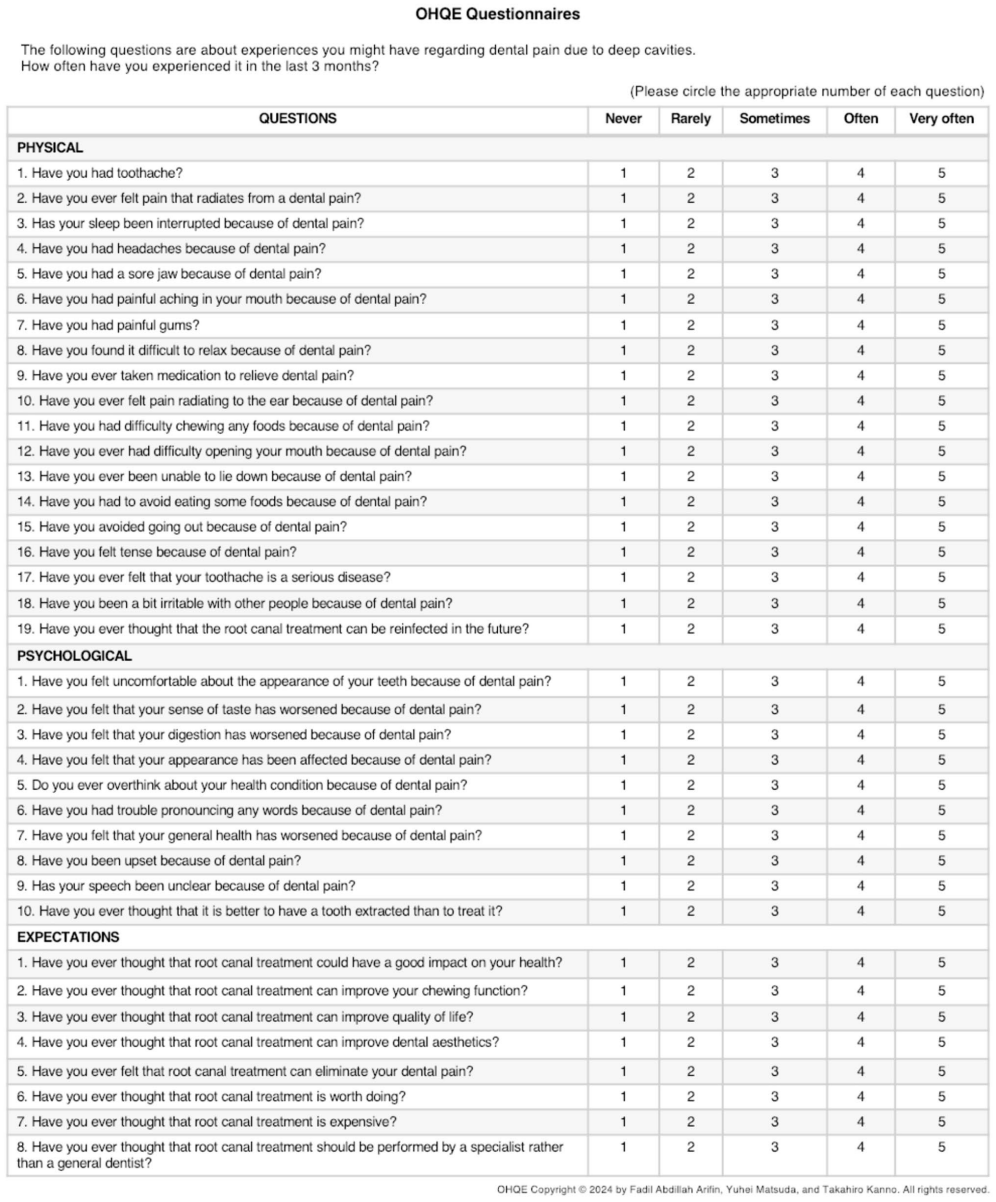
Oral health-related endodontic patient’s quality of life (OHQE) questionnaire

Each patient was asked to complete a 37-item OHQE questionnaire. The questions were evaluated using a five-point Likert scale, with each score indicating the following: 1, “never”; 2, “rarely”; 3, “sometimes”; 4, “often”; and 5, “very often.” Each subscale of the OHQE was rated on a scale of 1–5 (Fig. 2).

## Statistical analysis

The study calculated an approximate sample size prior to the start of the study. Based on previous literature [23], if we assumed a power of 0.8, a group ratio of 1:1, a difference in population means of 3, a standard deviation of 5.11, and an alpha error of 0.05 for the VAS pain score in the two groups, the sample size for both groups would be 47 cases. Assuming a dropout rate of 20%, the required sample size was calculated as 112 cases in the two groups.

The Shapiro–Wilk test was used to confirm data normality. Descriptive statistics were used to analyze the characteristics of the entire patient population, including percentages and mean values. The Mann–Whitney *U* and chi-square tests were used to compare baseline data between patients with irreversible pulpitis and those with pulp necrosis. A mixed-effects model was used to analyze the intervention effects, with the effect of time (before and after treatment and follow-up) as a fixed effect and the two diseases (irreversible pulpitis and pulp necrosis) as random effects. The Mann–Whitney *U* test was used to evaluate the difference in the mean score for each item of the OHQE scale before and after endodontic treatment between both dental pulp diseases. Statistical analyses were performed using SPSS (version 27; SPSS Japan Inc., Tokyo, Japan). A two-tailed *p* value was computed for each analysis. Significance was set at *p* < 0.05.

## Results

### Characteristics of the patients

Age and body mass index of patients with irreversible pulpitis (*n* = 62) and those with pulp necrosis (*n* = 69) were not significantly different (0.67 and 0.91, respectively). Females were predominant in both groups, without significant differences between the pulp disease groups (*p* = 0.92). Regarding the grade of caries, 59 cases of C3 (55 cases of irreversible pulpitis) and 72 cases of C4 (65 cases of pulp necrosis) were tabulated, with a significant difference between the groups (*p* < 0.01). The VAS score was significantly different between the pulp disease groups (*p* < 0.01) (Table 1).

**Table 1:** Patient characteristics before treatment

Variables	Categories	*n* (%) or mean [SD]		*p* value
		Irreversible pulpitis (*n* = 62)	Pulp necrosis (*n* = 69)	
Age (years)		36.5 [12.4]	35.9 [12.7]	0.67^a^
Sex	Male	23 (37.1)	25 (36.2)	0.92^b^
	Female	39 (62.9)	44 (63.8)	
Body mass index (kg/m^2^)		23.5 [3.6]	23.8 [4.3]	0.91^a^
Job	Employed	34 (54.8)	38 (55.1)	0.98^b^
	Unemployed	28 (45.2)	31 (44.9)	
University graduate	Yes	32 (51.6)	37 (53.6)	0.82^b^
	No	30 (48.4)	32 (46.4)	
Monthly income	< Rp. 3.300.000	43 (69.4)	50 (72.5)	0.70^b^
	≥ Rp. 3.300.000	19 (30.6)	19 (27.5)	
Number of housemates		4.2 [1.6]	4.5 [1.8]	0.29^a^
Systemic disease	Yes	15 (24.2)	10 (14.5)	0.16^b^
	No	47 (75.8)	59 (85.5)	
Medication taken	Yes	8 (12.9)	10 (14.5)	0.79^b^
	No	54 (87.1)	59 (85.5)	
Allergies	Yes	5 (8.1)	6 (8.7)	0.89^b^
	No	57 (91.9)	63 (91.3)	
Number of teeth		26.7 [4.3]	26.4 [4.6]	0.99^a^
Denture use	Yes	4 (6.4)	6 (8.7)	0.63^b^
	No	58 (93.6)	63 (91.3)	
Grade of caries	C3	55 (88.7)	4 (5.8)	< 0.01*^b^
	C4	7 (11.3)	65 (94.2)	
Grade of periodontal disease	S1	37 (59.7)	30 (43.5)	0.21^b^
	S2	21 (33.9)	29 (42.0)	
	S3	4 (6.5)	9 (13.0)	
	S4	0 (0.0)	1 (1.4)	
Brushing (times)		2.2 [0.5]	2.2 [0.6]	0.86^a^
VAS		4.5 [2.7]	2.7 [2.7]	< 0.01*^a^

*SD* standard deviation, *VAS* visual analogue scale

^a^Mann–Whitney *U* test

^b^Chi-square test ^*^p < 0.05

### Longitudinal repeated measures

**Fig. 3:**
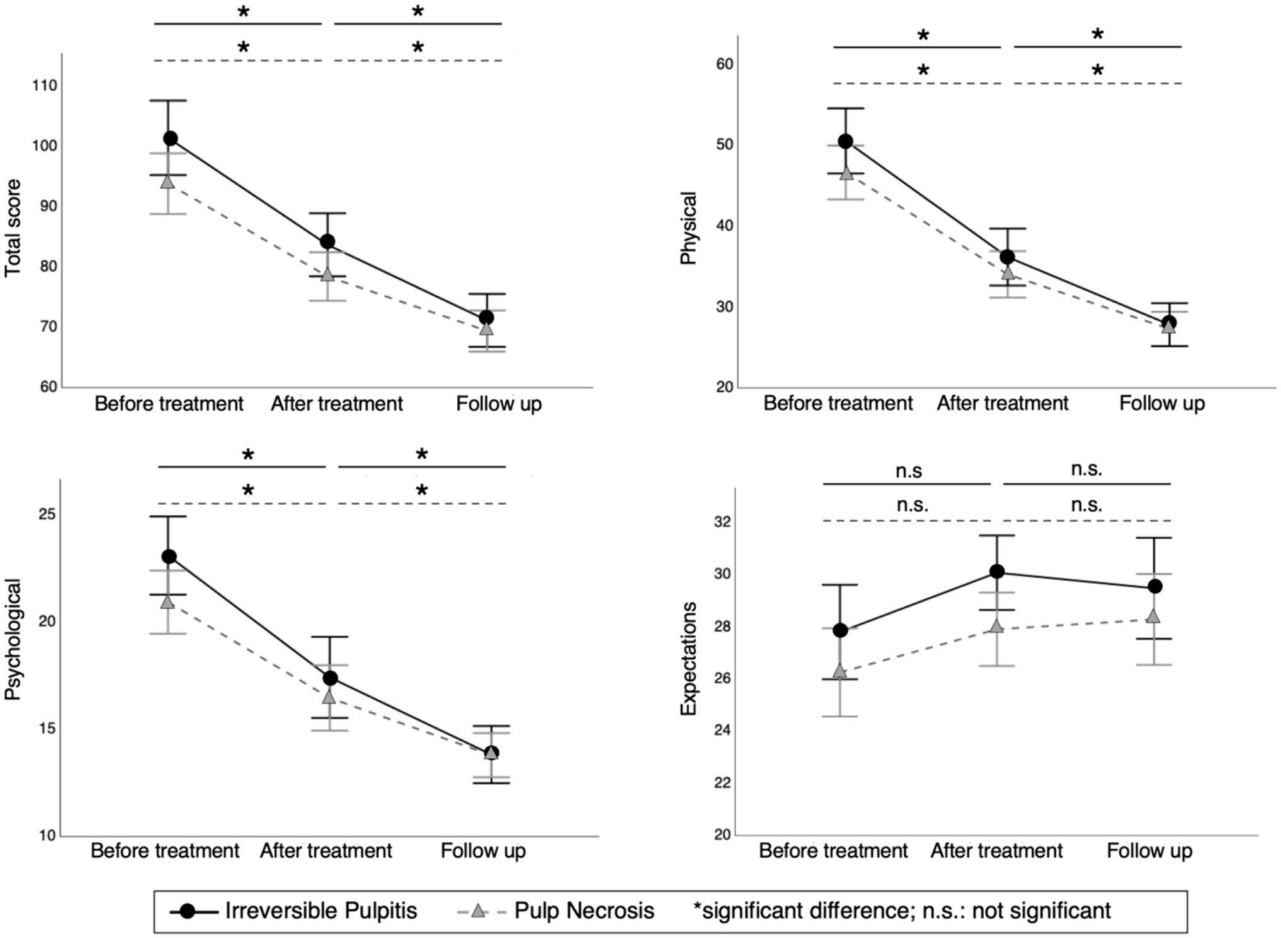
The mean of each parameter of the oral health-related endodontic patient’s quality of life (OHQE) score in the irreversible pulpitis and pulp necrosis groups

Table 2 presents the results of the mixed-effects model of the longitudinal repeated measures. Significant changes (*p* < 0.01) were seen in both groups from “before treatment” to “after treatment” in terms of the total score, physical, and psychological aspects. The expectations subscale showed no significant difference from “before treatment” to “after treatment” in both groups (*p* = 0.30 and *p* = 0.69, respectively). The interaction between irreversible pulpitis with “before treatment” and “after treatment” was not statistically significant for all subscales and for the total score. The total score, physical, and psychological aspects in both groups showed similar declining patterns on line graphs (Fig. 3). While the expectations line for pulp necrosis showed a slight rise, for irreversible pulpitis, the line declined slightly from “after treatment.”

**Table 2:** Linear mixed-effects model for changes in OHQE over time

Outcome/fixed effects	Estimate	Standard error	*p* value
Total score			
(Intercept)	69.41	2.32	< 0.01*
Irreversible pulpitis	1.76	3.37	0.60
Before treatment	24.46	2.86	< 0.01*
After treatment	9.04	2.86	< 0.01*
Irreversible pulpitis × before treatment interaction	5.86	4.15	0.16
Irreversible pulpitis × after treatment interaction	3.50	4.15	0.40
Physical			
(Intercept)	27.32	1.52	< 0.01*
Irreversible pulpitis	0.54	2.21	0.81
Before treatment	19.36	1.98	< 0.01*
After treatment	6.75	1.98	< 0.01*
Irreversible pulpitis × before treatment interaction	3.36	2.88	0.25
Irreversible pulpitis × after treatment interaction	1.60	2.88	0.58
Psychological			
(Intercept)	13.79	0.74	< 0.01*
Irreversible pulpitis	0.03	1.08	0.98
Before treatment	7.13	0.88	< 0.01*
After treatment	2.67	0.88	< 0.01*
Irreversible pulpitis × before treatment interaction	2.14	1.28	0.10
Irreversible pulpitis × after treatment interaction	0.93	1.28	0.47
Expectations			
(Intercept)	28.29	0.82	< 0.01*
Irreversible pulpitis	1.19	1.19	0.32
Before treatment	−2.03	0.93	0.30
After treatment	−0.38	0.93	0.69
Irreversible pulpitis × before treatment interaction	0.35	1.35	0.80
Irreversible pulpitis × after treatment interaction	0.97	1.35	0.47

^*^significant difference, *p* < 0.05

### Significant differences between groups for each item of the OHQE scale

Before endodontic treatment, both groups exhibited insignificant differences in all items for the expectations subscale (Table 3). Physical parameters showed a significant difference between irreversible pulpitis and pulp necrosis (numbers 8 and 14 in the OHQE questionnaire). Two items of the psychological aspect also showed a significant difference among the groups before treatment (numbers 2 and 3). Regarding post-endodontic treatment (Table 4), no physical items showed a significant difference between the two pulp diseases. Nevertheless, only one question showed a significant difference in the psychological subscale (number 10), as did two questions in the expectation subscale after endodontic treatment (numbers 5 and 6).

**Table 3:** The mean score of OHQE items before endodontic treatment between irreversible pulpitis and pulp necrosis group

OHQE items	Mean [SD]		*p* value
	Irreversible pulpitis (*n* = 62)	Pulp necrosis (*n* = 69)	
Physical			
1. Have you had a toothache?	3.2 [0.9]	3.1 [0.9]	0.85
2. Have you ever felt pain that radiates from a dental pain?	2.8 [1.3]	2.6 [1.7]	0.28
3. Has your sleep been interrupted because of dental pain?	2.8 [1.2]	2.7 [1.1]	0.54
4. Have you had headaches because of dental pain?	2.7 [1.3]	2.4 [1.2]	0.19
5. Have you had a sore jaw because of dental pain?	2.3 [1.3]	2.3 [1.1]	0.49
6. Have you had painful aching in your mouth because of dental pain?	2.7 [1.2]	2.4 [1.2]	0.25
7. Have you had painful gums?	2.7 [1.0]	2.9 [0.9]	0.43
8. Have you found it difficult to relax because of dental pain?	2.8 [1.2]	2.3 [1.1]	0.02*
9. Have you ever taken medication to relieve dental pain?	3.1 [1.1]	2.9 [1.1]	0.71
10. Have you ever felt pain radiating to the ear because of dental pain?	2.4 [1.3]	2.1 [1.2]	0.44
11. Have you had difficulty chewing any foods because of dental pain?	3.2 [1.0]	2.9 [1.0]	0.13
12. Have you ever had difficulty opening your mouth because of dental pain?	2.2 [1.1]	2.0 [1.2]	0.19
13. Have you ever been unable to lie down because of dental pain?	2.3 [1.1]	2.2 [1.1]	0.54
14. Have you had to avoid eating some foods because of dental pain?	3.2 [1.1]	2.7 [1.1]	0.03*
15. Have you avoided going out because of dental pain?	2.3 [1.2]	2.1 [1.0]	0.22
16. Have you felt tense because of dental pain?	2.5 [1.2]	2.1 [0.9]	0.06
17. Have you ever felt that your toothache is a serious disease?	2.7 [1.3]	2.4 [1.1]	0.13
18. Have you been a bit irritable with other people because of dental pain?	2.0 [0.9]	1.8 [0.9]	0.09
19. Have you ever thought that the root canal treatment can be reinfected in the future?	2.8 [0.9]	2.8 [1.1]	0.95
Psychological			
1. Have you felt uncomfortable about the appearance of your teeth because of dental pain?	2.3 [1.1]	2.1 [1.0]	0.51
2. Have you felt that your sense of taste has worsened because of dental pain?	2.3 [1.0]	1.8 [0.9]	< 0.01*
3. Have you felt that your digestion has worsened because of dental pain?	2.2 [1.0]	1.8 [0.9]	0.01*
4. Have you felt that your appearance has been affected because of dental pain?	2.5 [1.1]	2.1 [0.9]	0.07
5. Have you ever overthinking about your health condition because of dental pain?	2.3 [1.2]	2.2 [1.1]	0.84
6. Have you had trouble pronouncing any words because of dental pain?	2.2 [0.9]	2.0 [1.0]	0.14
7. Have you felt that your general health has worsened because of dental pain?	2.2 [1.0]	1.9 [0.9]	0.12
8. Have you been upset because of dental pain?	2.6 [1.0]	2.3 [0.9]	0.12
9. Has your speech been unclear because of dental pain?	2.0 [0.9]	1.9 [1.1]	0.19
10. Have you ever thought that it is better to have a tooth extracted than to treat it?	2.6 [1.2]	2.8 [1.7]	0.69
Expectations			
1. Have you ever thought that root canal treatment could have a good impact on your health?	3.9 [1.1]	3.6 [1.2]	0.12
2. Have you ever thought that root canal treatment can improve your chewing function?	3.2 [1.4]	3.2 [1.3]	0.97
3. Have you ever thought that root canal treatment can improve quality of life?	3.5 [1.2]	3.4 [1.3]	0.62
4. Have you ever thought that root canal treatment can improve dental aesthetics?	3.3 [1.4]	3.1 [1.3]	0.29
5. Have you ever felt that root canal treatment can eliminate your dental pain?	3.4 [1.3]	3.3 [1.2]	0.49
6. Have you ever thought that root canal treatment is worth doing?	3.7 [1.0]	3.4 [1.1]	0.17
7. Have you ever thought that root canal treatment is expensive?	3.3 [1.2]	2.9 [1.2]	0.09
8. Have you ever thought that root canal treatment should be performed by a specialist rather than a general dentist?	3.6 [1.3]	3.5 [1.3]	0.40

*SD* standard deviation ^*^p < 0.05

**Table 4:** The mean score of OHQE items after endodontic treatment between irreversible pulpitis and pulp necrosis group

OHQE items	Mean [SD]		*p* value
	Irreversible pulpitis (*n* = 62)	Pulp necrosis (*n* = 69)	
Physical			
1. Have you had a toothache?	2.4 [1.1]	2.1 [1.0]	0.11
2. Have you ever felt pain that radiates from a dental pain?	2.2 [0.9]	1.9 [0.9]	0.08
3. Has your sleep been interrupted because of dental pain?	1.8 [0.9]	1.7 [0.8]	0.98
4. Have you had headaches because of dental pain?	1.9 [0.9]	1.7 [0.9]	0.14
5. Have you had a sore jaw because of dental pain?	1.7 [0.9]	1.5 [0.7]	0.11
6. Have you had painful aching in your mouth because of dental pain?	1.9 [0.9]	1.7 [0.9]	0.15
7. Have you had painful gums?	2.2 [1.1]	1.9 [1.1]	0.18
8. Have you found it difficult to relax because of dental pain?	1.8 [0.9]	1.7 [0.8]	0.62
9. Have you ever taken medication to relieve dental pain?	2.3 [1.0]	2.0 [1.0]	0.24
10. Have you ever felt pain radiating to the ear because of dental pain?	1.6 [0.8]	1.6 [0.9]	0.75
11. Have you had difficulty chewing any foods because of dental pain?	2.0 [1.1]	1.9 [1.0]	0.83
12. Have you ever had difficulty opening your mouth because of dental pain?	1.7 [0.9]	1.6 [0.8]	0.45
13. Have you ever been unable to lie down because of dental pain?	1.7 [0.9]	1.7 [0.8]	0.98
14. Have you had to avoid eating some foods because of dental pain?	1.9 [1.0]	1.9 [0.9]	0.89
15. Have you avoided going out because of dental pain?	1.7 [0.9]	1.7 [0.9]	0.99
16. Have you felt tense because of dental pain?	1.8 [0.9]	1.6 [0.9]	0.19
17. Have you ever felt that your toothache is a serious disease?	1.8 [0.9]	1.9 [1.1]	0.87
18. Have you been a bit irritable with other people because of dental pain?	1.5 [0.7]	1.4 [0.7]	0.63
19. Have you ever thought that the root canal treatment can be reinfected in the future?	2.3 [1.0]	2.5 [1.1]	0.13
Psychological			
1. Have you felt uncomfortable about the appearance of your teeth because of dental pain?	1.8 [0.9]	1.7 [0.9]	0.21
2. Have you felt that your sense of taste has worsened because of dental pain?	1.7 [0.9]	1.5 [0.7]	0.21
3. Have you felt that your digestion has worsened because of dental pain?	1.6 [0.9]	1.5 [0.9]	0.50
4. Have you felt that your appearance has been affected because of dental pain?	1.9 [0.9]	1.8 [1.0]	0.64
5. Do you ever overthink about your health condition because of dental pain?	1.6 [0.9]	1.7 [1.0]	0.61
6. Have you had trouble pronouncing any words because of dental pain?	1.7 [0.9]	1.4 [0.7]	0.08
7. Have you felt that your general health has worsened because of dental pain?	1.7 [0.9]	1.6 [0.9]	0.36
8. Have you been upset because of dental pain?	1.9 [0.9]	1.6 [0.8]	0.11
9. Has your speech been unclear because of dental pain?	1.7 [1.1]	1.4 [0.7]	0.07
10. Have you ever thought that it is better to have a tooth extracted than to treat it?	1.9 [1.1]	2.3 [1.0]	0.03*
Expectations			
1. Have you ever thought that root canal treatment could have a good impact on your health?	4.1 [0.9]	3.8 [1.0]	0.12
2. Have you ever thought that root canal treatment can improve your chewing function?	3.7 [1.3]	3.6 [1.0]	0.08
3. Have you ever thought that root canal treatment can improve quality of life?	3.9 [0.9]	3.6 [1.0]	0.09
4. Have you ever thought that root canal treatment can improve dental aesthetics?	3.7 [1.1]	3.6 [1.1]	0.28
5. Have you ever felt that root canal treatment can eliminate your dental pain?	3.9 [0.9]	3.4 [1.1]	< 0.01*
6. Have you ever thought that root canal treatment is worth doing?	4.1 [0.9]	3.8 [0.9]	0.04*
7. Have you ever thought that root canal treatment is expensive?	2.9 [1.3]	2.8 [1.2]	0.31
8. Have you ever thought that root canal treatment should be performed by a specialist rather than a general dentist?	3.6 [1.1]	3.5 [1.3]	0.83

*SD* standard deviation ^*^p < 0.05

## Discussion

In this study, group comparisons showed no significant differences between the pulp disease groups at any time point. No interactions and no significant differences in changes over time were observed between the two groups. While both groups showed improvement of QoL over time after treatment, this did not differ between the two disease groups. In both groups, patients’ expectations of receiving endodontic treatment remained unchanged after treatment.

First, it is necessary to consider the generalizability of the results and the appropriateness of the comparison between patients with these pulp diseases. In this study, patients were included using the continuous rather than the random sampling method. Typically, a bias in the background factors of both groups was present; however, in this study, no significant differences in social, systemic, or oral health-related factors were found between groups. A slight difference in the average age and the sex of patients with the two dental pulp diseases in Indonesia was observed. However, Indonesia has a heterogeneous treatment environment: high-quality care is generally obtained in metropolitan areas, whereas the number of medical facilities in rural areas is limited [24]. Maintenance costs also vary between urban and rural areas, with urban areas generally being more expensive [25]. The data used in this study were collected from urban areas and therefore our findings may not be applicable to all endodontic patients. However, these results are likely similar to those of endodontic patients in developed countries.

Additionally, this study compared two pulp diseases for which the validity of the comparison between the two diseases had to be confirmed. Irreversible pulpitis usually occurs when infection or damage to nerves or blood vessels deep within the tooth is present. It is characterized by severe, persistent, and referred pain [22, 26]. In contrast, pulp necrosis is a condition in which the nerve tissue of the tooth has undergone necrosis (usually asymptomatic), tooth discoloration and halitosis are present, the infection has spread to the periapical tissue and surrounding bones, and tooth function is lost [16, 27, 28]. Endodontists often ignore the clinical manifestations experienced by patients with these two pulp diseases and then perform endodontic treatment without considering the patient’s QoL, and in particular the effects of these two pulp diseases. Therefore, the comparison made in this study is considered valid, and the results are meaningful.

In previous studies, irreversible pulpitis resulted in a decrease in QoL due to the pain experienced by patients, which was the main impetus for seeking emergency dental care [29]. Our results suggested that the response of QoL improvement on the physical and psychological subscales after endodontic treatment was higher for patients with irreversible pulpitis than for those with pulp necrosis. The decrease in psychological QoL caused by irreversible pulpitis is due to the orofacial referred pain, which reduces social activities of patients, resulting in absenteeism, dependence on analgesics, decreased ability to cope with dependents, lack of sleep, and difficulty in eating and speaking [30].

However, outcome parameters on the expectation parameters for endodontic treatment did not differ over time, between groups, or show interactions. Treatment of diseases with severe clinical symptoms, such as chronic pain, often increases patients’ expectations of the treatment outcomes [31]. Despite this, the treatment of irreversible pulpitis did not change patients’ expectations, possibly due to the lack of explanation by endodontists about the need, importance, and outcome of such treatment. In Indonesia, although inappropriate for medical and dental services, patients’ informed consent is often overlooked [32, 33]. Lack of communication between the dentist and the patient can lead to the patient lacking comprehension of the plan and treatment options [34]. Because immediate treatment is required, time constraints cause difficulty in communication between dentists and patients, as most of these dental treatments have a long duration [35]. Additionally, it is caused by a lack of awareness among dental healthcare providers, such as dentists and dental hygienists, of the importance of informed consent. This lack of awareness and the lack of patient education regarding care will result in patients’ distrust of dental providers [36]. An explanation of the endodontic treatment to be performed by endodontists is needed to increase patients’ expectations of endodontic treatment. Therefore, informed consent for patients with irreversible pulpitis is one of the determining factors by which endodontists can improve the patient’s QoL after endodontic treatment.

Pulp necrosis, which occurs when irreversible pulpitis is not treated properly, is the final stage in the pathological course of caries. In the early stages, symptoms may include severe pain due to pressure caused by pulp inflammation and signs of nerve hypersensitivity around the inflamed area [22, 37]. If not properly treated, the infection can spread to the gingiva and around the face, causing swelling [16]. Because pulp necrosis is an advanced form of irreversible pulpitis, it is plausible that the OHQE scores were lower in this group than in the irreversible pulpitis group at all time points. Additionally, one of the characteristics of advanced pulp necrosis is that it is asymptomatic [27]. Because the OHQE used in this study was an OHRQoL scale that focused specifically on endodontic pain, the significantly low OHQE scores in the pretreatment phase likely reflected the presence of pain. However, the results of this study did not show significant differences between irreversible pulpitis and pulp necrosis groups in the pretreatment phase; thus, patients with relatively symptomatic pulp necrosis may also have been included. Therefore, although endodontic treatment methods for pulp necrosis do not differ significantly, attention should be paid equally to patients with or without symptoms, and treatment explanations and informed consent should be provided to meet patient expectations for endodontic treatment.

Endodontic treatment is effective for irreversible pulpitis and pulp necrosis, followed by prosthetic treatment performed after removal of the infected tissue and subsequent root canal filling [38]. However, endodontic treatment for pulpal necrosis in this study generally required a longer treatment duration than that for irreversible pulpitis. A longer treatment duration is expected to increase the opportunity for dentists to communicate and to provide explanations regarding the treatment to patients. However, an extension of the treatment duration can lead to a decrease in the patient’s QoL, such as an increase in the effort required for the patient to visit the hospital or the occurrence of pain during treatment [39]. In this study, our statistical model investigated an interaction between time and pathological factors, but found none. Therefore, it is important to provide effective questions to endodontic patients, as well as shorten the duration of the endodontic treatment.

The digestive health and sense of taste issues found in this study are concerns that should be addressed by the endodontist before treatment, as both of these can be associated with decreased appetite. The related questions were “Have you felt that your sense of taste has worsened because of dental pain?” and “Have you felt that your digestion has worsened because of dental pain?” The results of the current study are acceptable, as previous research has also indicated that untreated dental caries can lead to both irreversible pulpitis and pulp necrosis, resulting in decreased appetite [40]. Both groups may experience a reduction in appetite because of pain symptoms that cause distress during digestion and tasting. Therefore, endodontists should focus on monitoring the dietary intake of patients with irreversible pulpitis and pulp necrosis to preserve the patient’s digestive health. Furthermore, endodontists also need to pay attention to patient expectations regarding pain reduction after endodontic treatment using the question “Have you ever felt that root canal treatment can eliminate your dental pain?” A previous study showed that immediate treatment of symptomatic irreversible pulpitis or apical periodontitis was more successful in reducing pain [41]. Thus, the results of this study are acceptable. Expectation scores were reportedly lower in patients with pulp necrosis than in those with irreversible pulpitis. The high expectations of patients with irreversible pulpitis in this study may stem from the fact that they endured severe pain before treatment and achieved instant post-treatment pain relief.

This study had a few limitations. First, in this study, data on the periapical status of patients were not obtained. Therefore, all patients likely to have periapical disease may have been included in the classification of patients with pulp necrosis. Second, no data were available on the duration of endodontic treatment in each patient, considering that the need for the duration of endodontic treatment in each patient varies. Third, there were no data on the level of dental health literacy in each patient, which could affect the research results. Lastly, the current study reflected cases in which only one tooth required endodontic treatment, rather than the complete oral health status of the patients. These limitations can introduce bias into the research, resulting in potentially inaccurate findings. In future, prospective observational studies on a larger scale are required to overcome these limitations, reduce the prevalence of bias in research, and ensure that the findings are more representative of the whole population. Additionally, more endodontists are likely to become aware of the subjective judgments of their patients as better research results are obtained.

In conclusion, we found no significant difference in QoL enhancement by endodontic treatment between the two pulp diseases. The physical and psychological subscale scores of the OHQE of each group improved over time. Conversely, patients’ expectations regarding endodontic treatment remained unchanged after treatment. These results indicated that patients continue to lack comprehension of the importance of the endodontic treatment that they have been receiving. Hence, dental providers, particularly endodontists, should contemplate elucidating the significance of endodontic treatment for patients and address the factors that enhance patient expectations.

## Data Availability

The data to support the findings of this study will be available from the corresponding author upon reasonable request.

## References

[1] Fransson H, Dawson V. Tooth survival after endodontic treatment. Int Endod J. 2023;56(Suppl 2):140–53. 10.1111/iej.13835. 36149887 10.1111/iej.13835

[2] Tsesis I, Taschieri S, Slutzky-Goldberg I. Contemporary endodontic treatment. Int J Dent. 2012;2012: 231362. 10.1155/2012/231362. 23209468 10.1155/2012/231362 PMC3502872

[3] Liu P, McGrath C, Cheung GSP. Quality of life and psychological well-being among endodontic patients: a case-control study. Aust Dent J. 2012;57:493–7. 10.1111/j.1834-7819.2012.01722.x. 23186576 10.1111/j.1834-7819.2012.01722.x

[4] Gerritsen AE, Allen PF, Witter DJ, Bronkhorst EM, Creugers NH. Tooth loss and oral health-related quality of life: a systematic review and meta-analysis. Health Qual Life Outcomes. 2010;8:126. 10.1186/1477-7525-8-126. 21050499 10.1186/1477-7525-8-126 PMC2992503

[5] Liu P, McGrath C, Cheung GS. Improvement in oral health-related quality of life after endodontic treatment: a prospective longitudinal study. J Endod. 2014;40:805–10. 10.1016/j.joen.2014.02.008. 24862707 10.1016/j.joen.2014.02.008

[6] Alroudhan IE, Ravi J, Magar SS, Alam MK, Alsharari KN, Alsharari FM. Oral health-related quality of life and satisfaction after root canal treatment according to operator expertise: a longitudinal prospective study. Saudi Endod J. 2021;11:388. 10.4103/sej.sej_291_20.

[7] Hamasha AA, Hatiwsh A. Quality of life and satisfaction of patients after nonsurgical primary root canal treatment provided by undergraduate students, graduate students and endodontic specialists. Int Endod J. 2013;46:1131–9. 10.1111/iej.12106. 23560436 10.1111/iej.12106

[8] Eckerbom M, Flygare L, Magnusson T. A 20-year follow-up study of endodontic variables and apical status in a swedish population. Int Endod J. 2007;40:940–8. 10.1111/j.1365-2591.2007.01290.x. 17883402 10.1111/j.1365-2591.2007.01290.x

[9] Kirkevang LL, Vaeth M, Horsted-Bindslev P, Wenzel A. Longitudinal study of periapical and endodontic status in a Danish population. Int Endod J. 2006;39:100–7. 10.1111/j.1365-2591.2006.01051.x. 16454789 10.1111/j.1365-2591.2006.01051.x

[10] Gomes BPFDA, Herrera DR. Etiologic role of root canal infection in apical periodontitis and its relationship with clinical symptomatology. Braz Oral Res. 2018;32:82–110. 10.1590/1807-3107bor-2018.vol32.0069. 10.1590/1807-3107bor-2018.vol32.0069 30365610

[11] Modaresi J, Davoudi A, Badrian H, Sabzian R. Irreversible pulpitis and achieving profound anesthesia: complexities and managements. Anesth Essays Res. 2016;10:3–6. 10.4103/2F0259-1162.164675. 26957681 10.4103/0259-1162.164675 PMC4767074

[12] Petrini M, Ferrante M, Ciavarelli L, Brunetti L, Vacca M, Spoto G. Prostaglandin E2 to diagnose between reversible and irreversible pulpitis. Int J Immunopathol Pharmacol. 2012;25:157–63. 10.1177/039463201202500118. 22507328 10.1177/039463201202500118

[13] Cimilli H, Karacayli U, Şişman N, Kartal N, Mumcu G. Comparison of the oral health-related quality of life and dental pain in symptomatic irreversible pulpitis and pericoronitis. J Dent Sci. 2012;7:250–60. 10.1016/j.jds.2012.05.014.

[14] López-Marcos JF. Aetiology, classification and pathogenesis of pulp and periapical disease. Med Oral Patol Oral Cir Bucal. 2004;9(Suppl):52–62. 15580137

[15] Koç S, Del-Fabbro M. Does the etiology of pulp necrosis affect regenerative endodontic treatment outcomes? a systematic review and meta-analyses. J Evid Based Dent Pract. 2020;20: 101400. 10.1016/j.jebdp.2020.101400. 32381409 10.1016/j.jebdp.2020.101400

[16] Mejare IA, Axelsson S, Davidson T, Frisk F, Hakeberg M, Kvist T, Norlund A, Petersson A, Portenier I, Sandberg H, Tranaeus S, Bergenholtz G. Diagnosis of the condition of the dental pulp: a systematic review. Int Endod J. 2012;45:597–613. 10.1111/j.1365-2591.2012.02016.x. 22329525 10.1111/j.1365-2591.2012.02016.x

[17] Nogueira NG, Lima MDDM, Moura JSS, Lima CCB, Moura MSD, Castro MVVS, Moura LDFADD. Impact of pulp necrosis on oral health-related quality of life of children with early childhood caries. J Dent Child (Chic). 2022;89:11–7. 35337394

[18] Arifin FA, Matsuda Y, Kanno T. Development and validation of oral health-related quality of life scale for patients undergoing endodontic treatment (OHQE) for irreversible pulpitis. Healthcare. 2023;11:2859. 10.3390/healthcare11212859. 37958003 10.3390/healthcare11212859 PMC10648889

[19] Huh J, Nam H, Kim J, Park J, Shin S, Lee R. Studies of automatic dental cavity detection system as an auxiliary tool for diagnosis of dental caries in digital x-ray image. PMP. 2015;26:52–8.

[20] Caton JG, Armitage G, Berglundh T, Chapple ILC, Jepsen S, Kornman KS, Mealey BL, Papapanou PN, Sanz M, Tonetti MS. A new classification scheme for periodontal and peri-implant diseases and conditions—introduction and key changes from the 1999 classification. J Clin Periodontol. 2018;45:S1–8. 10.1111/jcpe.12935. 29926489 10.1111/jcpe.12935

[21] Guide to clinical endodontics. American association of endodontists. 2013. https://www.aae.org/specialty/clinical-resources/guide-clinical-endodontics/. Accessed 23 Mar 2024.

[22] Endodontics: colleagues for excellence. In: endodontic diagnosis. American association of endodontists. 2013. https://www.aae.org/specialty/wp-content/uploads/sites/2/2017/07/endodonticdiagnosisfall2013.pdf. Accessed 23 Mar 2024.

[23] Shahi S, Asghari V, Rahimi S, Lotfi M, Samiei M, Yavari H, Shakouie S, Nezafati S. Postoperative pain after endodontic treatment of asymptomatic teeth using rotary instruments: a randomized clinical trial. Iran Endod J. 2016;11:38–43. 10.22037/iej.v11i1.9408. 26843876 10.7508/iej.2016.01.008 PMC4731532

[24] Noya F, Carr S, Thompson S, Clifford R, Playford D. Factors associated with the rural and remote practice of medical workforce in maluku islands of Indonesia: a cross-sectional study. Hum Resour Health. 2021;19:126. 10.1186/s12960-021-00667-z. 34627282 10.1186/s12960-021-00667-z PMC8502290

[25] Shao Q, Tao R, Luca MM. The effect of urbanization on health care expenditure: evidence from China. Front Public Health. 2022;10:850872. 10.3389/2Ffpubh.2022.850872. 35242736 10.3389/fpubh.2022.850872 PMC8885621

[26] Raoof M, Vazavandi E, Parizi MT, Hatami N, Mohammadalizadeh S, Amanpour S, Haghani J. Clinical, radiological, and histological correlation in diagnosis of pulpitis. Dent Res J (Isfahan). 2022;19:25. 35432790 PMC9006151

[27] Kontakiotis EG, Filippatos CG, Stefopoulos S, Tzanetakis GN. A prospective study of the incidence of asymptomatic pulp necrosis following crown preparation. Int Endod J. 2015;48:512–7. 24964352 10.1111/iej.12340

[28] Moccelini BS, de Alencar NA, Bolan M, Magno MB, Maia LC, Cardoso M. Pulp necrosis and crown discoloration: a systematic review and meta-analysis. Int J Paediatr Dent. 2018;28:432–42. 10.1111/ipd.12372. 10.1111/ipd.12372 29896799

[29] Edwards D, Rasaiah S, Ahmed SH, Breckons M, Stone SJ, Currie CC, Durham J, Whitworth J. The financial and quality of life impact of urgent dental presentations: a cross-sectional study. Int Endod J. 2023;56:697–709. 10.1111/iej.13917. 36975836 10.1111/iej.13917

[30] Edwards D, Bailey O, Stone SJ, Duncan H. How is carious pulp exposure and symptomatic irreversible pulpitis managed in UK primary dental care? Int Endod J. 2021;54:2256–75. 10.1111/iej.13628. 34487553 10.1111/iej.13628

[31] Wiering B, de Boer D, Krol M, Wieberneit-Tolman H, Delnoij D. Entertaining accurate treatment expectations while suffering from chronic pain: an exploration of treatment expectations and the relationship with patient—provider communication. BMC Health Serv Res. 2018;18:706. 10.1186/s12913-018-3497-8. 30200955 10.1186/s12913-018-3497-8 PMC6131883

[32] Herwanda H, Rahmayani L, Fadhilla S. Gambaran penggunaan persetujuan tindakan medis (informed consent) oleh dokter gigi muda di RSGM UNSYIAH. Cakradonya Dent J. 2016;8:123–31.

[33] Nengrum ZS, EniyatiZakhariasIka EKPFA. Tinjauan pelaksanaan informed concent pemeriksaan kontras colon in loop di instalasi radiologi RSUD kota yogyakarta. Jurnal Indonesia Sehat. 2022;1:216–23.

[34] Kakar H, Gambhir RS, Singh S, Kaur A, Nanda T. Informed consent: corner stone in ethical medical and dental practice. J Fam Med Prim Care. 2014;3:68–71. 10.4103/2F2249-4863.130284. 10.4103/2249-4863.130284 PMC4005206 24791241

[35] Maria A, Anna K, Adreas K, Ioannis T. Basic tips for communicating with a new dental patient. ARC J Dent Sci. 2016;1:4–11. 10.20431/2456-0030.0104002.

[36] Murphy M, McCaughan E, Thompson G, Carson MA, Hanna JR, Donovan M, Wilson RH, Fitzsimons D. Trusting relationships between patients with non-curative cancer and healthcare professionals create ethical obstacles for informed consent in clinical trials: a grounded theory study. BMC Palliat Care. 2023;22:85. 10.1186/s12904-023-01204-6. 37393250 10.1186/s12904-023-01204-6 PMC10315048

[37] Gemmell A, Stone S, Edwards D. Investigating acute management of irreversible pulpitis: a survey of general dental practitioners in North East England. Br Dent J. 2020;228:521–6. 10.1038/s41415-020-1419-8. 32277210 10.1038/s41415-020-1419-8

[38] Bjørndal L, Simon S, Tomson PL, Duncan HF. Management of deep caries and the exposed pulp. Int Endod J. 2019;52:949–73. 10.1111/iej.13128. 30985944 10.1111/iej.13128

[39] Konieczny M, Cipora E, Roczniak W, Babuśka-Roczniak M, Wojtaszek M. Impact of time to initiation of treatment on the quality of life of women with breast cancer. Int J Environ Res Public Health. 2020;17:8325. 10.3390/ijerph17228325. 33187071 10.3390/ijerph17228325 PMC7696805

[40] Gunay B, Kaya MS, Ozgen IT, Guler EM, Kocyigit A. Evaluation of the relationship between pain inflammation due to dental caries and growth parameters in preschool children. Clin Oral Invest. 2023;27:3721–30. 10.1007/s00784-023-04988-2. 10.1007/s00784-023-04988-2 PMC10088690 37036512

[41] Alhilou AM, Al-Moraissi EA, Bakhsh A, Christidis N, Näsman P. Pain after emergency treatments of symptomatic irreversible pulpitis and symptomatic apical periodontitis in the permanent dentition: a systematic review of randomized clinical trials. Front Oral Health. 2023;4:1147884. 10.3389/froh.2023.1147884. 37920592 10.3389/froh.2023.1147884 PMC10618681

